# Effects of alcohol consumption, cigarette smoking, and betel quid chewing on upper digestive diseases: a large cross-sectional study and meta-analysis

**DOI:** 10.18632/oncotarget.20831

**Published:** 2017-09-11

**Authors:** Yun-Shiuan Chuang, Meng-Chieh Wu, Fang-Jung Yu, Yao-Kuang Wang, Chien-Yu Lu, Deng-Chyang Wu, Chie-Tong Kuo, Ming-Tsang Wu, I-Chen Wu

**Affiliations:** ^1^ Department of Public Health, College of Health Sciences, Kaohsiung Medical University, Kaohsiung, Taiwan; ^2^ Division of Gastroenterology, Department of Internal Medicine, Kaohsiung Medical University Hospital, Kaohsiung, Taiwan; ^3^ Department of Internal Medicine, Kaohsiung Municipal Ta-Tung Hospital, Kaohsiung, Taiwan; ^4^ Faculty of Medicine, Department of Medicine, College of Medicine, Kaohsiung Medical University, Kaohsiung, Taiwan; ^5^ Department of Physics, National Sun Yat-sen University, Kaohsiung, Taiwan; ^6^ Department of Family Medicine, Kaohsiung Medical University Hospital, Kaohsiung, Taiwan; ^7^ Research Center for Environmental Medicine, Kaohsiung Medical University, Kaohsiung, Taiwan

**Keywords:** smoking, alcohol, betel, reflux esophagitis, esophageal cancer

## Abstract

Cigarette smoking is a well-known risk factor of upper digestive diseases. Findings on alcohol's effect on these diseases are inconsistent and with the exception of its association with esophageal cancer, little is known about betel quid chewing. This study investigated the association between use of these three substances and upper digestive diseases. We collected data from 9,275 patients receiving upper endoscopies between April 2008 and December 2013. Polynomial regressions were used to analyze the association between risk factors and diseases of the esophagus, stomach and duodenum. Meta-analysis for use of these substances and esophageal diseases was also performed. Participants who simultaneously consumed cigarettes, alcohol and betel quid had a 17.28-fold risk of esophageal cancer (95% CI = 7.59–39.33), 2.99-fold risk of Barrette's esophagus (95% CI = 2.40–4.39), 1.60-fold risk of grade A-B erosive esophagitis (95% CI = 1.29–2.00), 2.00-fold risk of gastric ulcer (95% CI = 1.52–2.63), 2.12-fold risk of duodenitis (95% CI = 1.55–2.89) and 1.29-fold risk of duodenal ulcer (95% CI = 1.01–1.65). Concurrent consumption of more substances was associated with significantly higher risk of developing these diseases. Meta-analysis also revealed use of the three substances came with a high risk of esophageal diseases. In conclusions, cigarette smoking, alcohol drinking and betel quid chewing were associated with upper digestive tract diseases.

## INTRODUCTION

There are over one billion smokers in the world and among them, four million will die as a result of their habit [1]. Alcohol consumption is estimated to cause more than ten percent of all deaths in the European Union [2], and betel quid (area nut), another addictive substance used by approximately 600 million people in Asia, has been associated with oral and esophageal cancers [3]. Thus, the adverse health effects caused by these addictive substance have become an important public health problem in the world.

Cigarette smoking causes different illnesses, including cardiovascular disease, pulmonary disease and malignancy [4]. It has also been associated with diseases of the upper digestive tract, including peptic ulcer disease [5, 6] and has been found to influence the rate of healing in patients with gastric ulcers [7]. Excessive alcohol consumption has also been associated with diseases with high mortality rates, including liver cirrhosis, pancreatic disease and various cancers [8]. Ethanol has been found to induce lesions in gastric mucosa [9]. The combined use of cigarette smoking and alcohol consumption is a known risk factor for erosive esophagitis and Barrett's esophagus in Japan [10, 11].

Gastric cancer and esophageal cancer are the fifth and eighth most common cancers in the world [12] and the seventh and ninth most deadly cancers in Taiwan [13]. Worldwide, over 700,000 and 400,000 people died from gastric and esophageal cancers, respectively, in 2008 [12]. Two large cohort studies, one from the USA and the other from the Netherlands, found cigarette smoking increased the risk for esophageal adenocarcinoma, esophageal squamous cell carcinoma, and gastric adenocarcinoma while alcohol increased the risk of esophageal squamous cell carcinoma [14, 15]. Another large cohort study from Lithuania found heavy alcohol intake to be a risk factor in gastric cancer in men [16]. In Taiwan, betel quid chewing is an independent risk factor for esophageal cancer [17–20] and gastric cancer [21]. To the best of our knowledge, no study has investigated the effect of all three substances (alcohol, cigarette and betel quid) on the development of different upper digestive diseases.

Thus, in this large cross-sectional study analyzing data collected from questionnaire and upper endoscopic examinations administered to participants at four hospitals in southern Taiwan from April 2008 to December 2013, we investigated the association between usage of alcohol, cigarette and betel quid and the diagnosis and severity of upper digestive disease and malignancy. We also conducted a meta-analysis of studies examining the impact of use of these three substances on the development of esophageal diseases.

## RESULTS

**Figure 1:**
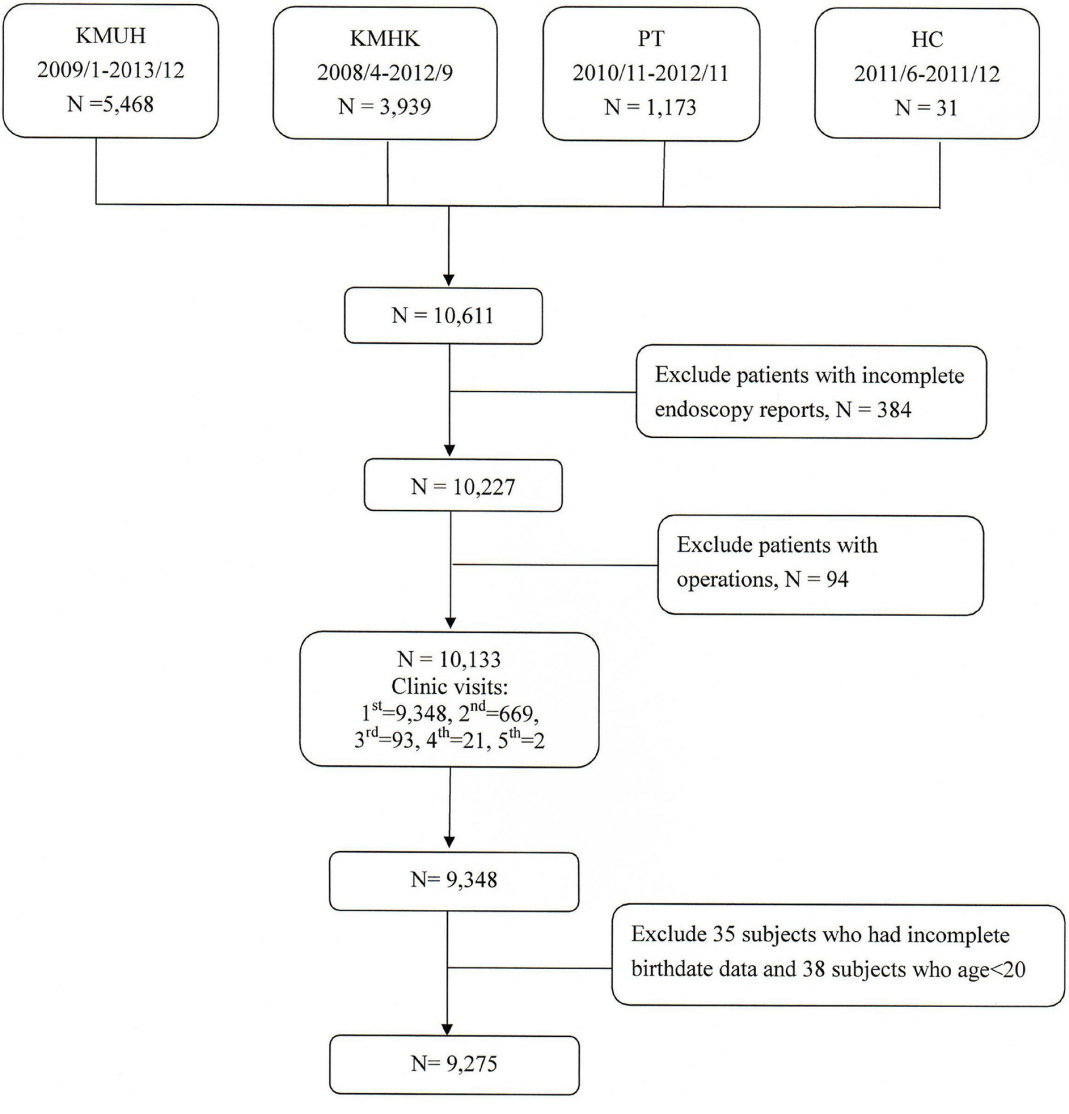
Study flowchart Abbreviations: KMUH, Kaohsiung Medical University Hospital; KMHK, Kaohsiung Municipal Hsiao-Kang Hospital; PT, Ministry of Health and Welfare Ping-Tung Hospital; HC, Ministry of Health and Welfare Heng-Chun Tourism Hospital.

A total of 9,275 participants received upper endoscopies at the four hospitals between April 2008 and December 2013 (Figure 1). Because only thirty-one received these examinations at Heng-Chun Tourism Hospital (HC), their data were combined with the data collected at nearby Ping-Tung Hospital (PT). Thus, our analysis is based on three sets of data from 4,896 (52.8%), 3,541 (38.2%) and 838 (9.0%) participants at Kaohsiung Medical University Hospital (KMUH), Kaohsiung Municipal Hsiao-Kang Hospital (KMHK), and PT, respectively (Supplementary Table 1). As can be seen in Table 1, most participants (48.38%) were 50–69 years old and most (55.47%) had a normal body mass index (BMI). Almost twelve percent (11.71%) smoked cigarettes only, 3.27% drank alcohol only, and 0.4% used betel quid only. Around five percent (5.15%) habitually used all three substances.

**Table 1:** Baseline characteristics of 9,275 patients receiving endoscopic examinations

	*N*	%
**Gender**		
Female	4710	50.78
Male	4565	49.22
**Age**		
20–49	3442	37.11
50–69	4487	48.38
70–97	1346	14.51
**BMI**		
< 18.5, underweight	636	6.86
18.5–24.9, normal	5145	55.47
> 25, overweight	3494	37.67
**Substance use**		
None	6601	71.17
Cigarettes	1086	11.71
Alcohol	303	3.27
Betel quid	37	0.4
Cigarettes+Alcohol	551	5.94
Cigarettes+Betel quid	181	1.95
Alcohol+Betel quid	38	0.41
Cigarettes+Alcohol+Betel quid	478	5.15

The distribution of disease was analyzed by endoscopic findings and pathological reports. Analysis of both reports found that 32.25% of all participants had gastroesophageal reflux (GERD) A-B, 4.49% had Barrett's esophagus and 0.55% had esophageal cancer. 11.36% were diagnosed with gastric ulcer and 0.60% diagnosed with gastric adenocarcinoma. 18.19% of all participants were diagnosed as having duodenal ulcers, 6.71% duodenitis and 0.02% tumors of the duodenum (Table 2).

**Table 2:** Clinicopathological characteristics of 9,275 patients at different positions

	Number	%
**Esophagus**		
Normal	5569	60.04
GERD A-B	2991	32.25
GERD C-D	12	0.13
Barrett's esophagus	416	4.49
Esophageal cancer	51	0.55
Other (ulcer, Mallory-Weiss tear, EV and papilloma)	236	2.54
**Stomach**		
Normal	7993	86.18
GU	1054	11.36
Gastric polyp	8	0.09
Gastric adenocarcinoma	56	0.60
GIST, lymphoma, MALToma	97	1.05
Other (GV and other cancer)	67	0.72
**Duodenum**		
Normal	6964	75.08
Duodenitis	622	6.71
DU	1687	18.19
Tumor	2	0.02

Abbreviations: GERD, gastroesophageal reflux disease EV, esophageal varices; GU, gastric ulcer; GIST, Gastrointestinal Stromal Tumor; MALToma, Mucosa-associated lymphoid tissue *lymphoma;* GV, gastric varices; DU, duodenal ulcer.

Tables 3 to 5 analyze the risk factors for esophagus, stomach and duodenum disease, respectively. In general, males were at higher risk than females for any of the diseases. Increased age was a risk factor for grade A-B reflux esophagitis, Barrette's esophagus, gastric ulcer and duodenal ulcer. Compared with the normal BMI (18.5–24.9) group, overweight subjects (BMI>25) were at significantly higher risk of reflux esophagitis (adjusted odds ratio [aOR]: 1.41, 95% CI: 1.28–1.55), Barrett's esophagus (aOR: 1.39, 95% CI: 1.13–1.71) and gastric ulcer (aOR: 1.25, 95% CI: 1.09–1.43). Underweight subjects (BMI < 18.5) were at significantly lower risks for reflux esophagitis (aOR: 0.73, 95% CI: 0.60–0.89) and duodenal ulcer (aOR: 0.71, 95% CI: 0.56–0.91). Patients diagnosed with cancers of the esophagus and stomach had significantly lower BMIs. The major histological type of esophageal cancer was squamous cell carcinoma (98.04%), while adenocarcinoma accounted for 1.96%.

**Table 3:** Risk factors for developing esophagus disease in 9,275 patients

	GERD A-B						GERD C-D						Barrett's esophagus						Esophageal cancer					
	cOR^1^	95%CI		aOR^1^	95%CI		cOR^1^	95%CI		aOR^1^	95%CI		cOR^1^	95%CI		aOR^1^	95%CI		cOR^1^	95%CI		aOR^1^	95%CI	
Gender																								
Female	1			1			1			1			1			1			1			1		
Male	1.97	1.80	2.16	1.65	1.48	1.82	2.80	0.84	9.32	1.25	0.29	5.31	2.92	2.36	3.61	1.97	1.54	2.52	22.42	6.98	72.07	9.18	2.56	32.86
Age																								
20-49	1			1			1			1			1			1			1			1		
50-69	1.14	1.03	1.25	1.10	1.00	1.22	0.65	0.18	2.43	0.67	0.18	2.54	1.26	1.00	1.59	1.25	0.99	1.58	2.60	1.32	5.12	2.95	1.47	5.93
70-97	1.28	1.12	1.47	1.28	1.11	1.47	1.79	0.43	7.49	2.18	0.51	9.39	2.18	1.65	2.88	2.31	1.74	3.08	1.35	0.47	3.90	1.86	0.63	5.53
BMI																								
<18.5, underweight	0.68	0.56	0.82	0.73	0.60	0.89	1.80	0.20	16.15	1.89	0.21	17.24	0.62	0.38	1.01	0.70	0.43	1.15	2.83	1.40	5.73	4.36	2.06	9.23
18.5-24.9, normal	1			1			1			1			1			1			1			1		
>25, overweight	1.52	1.38	1.67	1.41	1.28	1.55	3.06	0.90	10.48	2.79	0.81	9.63	1.57	1.28	1.92	1.39	1.13	1.71	0.75	0.38	1.48	0.52	0.26	1.03
Substance use																								
None	1			1			1			1			1			1			1			1		
Cigarettes	1.62	1.41	1.86	1.27	1.09	1.47	4.51	1.08	18.93	3.79	0.76	18.84	2.13	1.60	2.85	1.57	1.15	2.14	1.37	0.30	6.18	0.66	0.14	3.06
Alcohol	1.44	1.12	1.85	1.13	0.87	1.46	5.28	0.61	45.47	5.04	0.53	48.30	1.96	1.17	3.29	1.50	0.89	2.55	9.60	3.03	30.48	5.66	1.71	18.73
Betel quid	1.58	0.77	3.23	1.27	0.62	2.62	-	-	-	-	-	-	2.08	0.48	9.00	1.57	0.36	6.87	-	-	-	-	-	-
Cigarettes+Alcohol	1.81	1.50	2.19	1.38	1.13	1.68	3.30	0.39	28.38	2.86	0.29	28.20	2.96	2.07	4.22	2.12	1.46	3.10	15.02	6.32	35.68	6.73	2.69	16.84
Cigarettes+Betel quid	1.88	1.36	2.61	1.31	0.94	1.83	10.70	1.24	92.59	9.01	0.86	94.69	3.97	2.31	6.81	2.67	1.53	4.67	14.58	3.99	53.28	6.59	1.72	25.23
Alcohol+Betel quid	1.82	0.92	3.62	1.29	0.64	2.58	-	-	-	-	-	-	2.08	0.48	9.00	1.46	0.33	6.38	21.61	2.65	176.23	12.35	1.45	105.17
Cigarettes+Alcohol+Betel quid	2.27	1.84	2.79	1.60	1.29	2.00	4.63	0.54	39.79	3.91	0.38	40.64	4.34	3.04	6.21	2.99	2.04	4.39	42.04	19.85	89.04	17.28	7.59	39.33
p value for trend				<0.0001						0.0227						<0.0001						<0.0001		

Abbreviations: cOR, crude odds ratio; aOR, adjusted odds ratio; CI, confidence interval.

^1^Using esophagus normal as reference category. Adjusted odds ratio were adjusted for gender, age, BMI and substance use.

Bold indicates statistical significance.

**Table 4:** Risk factors for developing stomach disease in 9,275 patients

	GU						Gastric adenocarcinoma					
	cOR^1^	95%CI		aOR1	95% CI		cOR^1^	95% CI		aOR^1^	95% CI	
**Gender**												
Female	**1**			1			1			1		
Male	**1.43**	**1.25**	**1.62**	1.06	0.91	1.23	1.54	0.90	2.62	1.34	0.72	2.49
**Age**												
20–49	1			1			1			1		
50–69	**2.52**	**2.14**	**2.98**	**2.54**	**2.14**	**3.00**	**4.78**	**1.84**	**12.38**	**5.12**	**1.97**	**13.31**
70–97	**3.80**	**3.12**	**4.63**	**4.00**	**3.28**	**4.90**	**14.19**	**5.38**	**37.41**	**15.09**	**5.68**	**40.11**
**BMI**												
< 18.5, underweight	0.76	0.56	1.03	0.85	0.62	1.15	1.98	0.91	4.33	**2.33**	**1.06**	**5.13**
18.5–24.9, normal	1			1			1			1		
> 25, overweight	1.35	1.18	1.54	1.25	**1.09**	**1.43**	0.77	0.42	1.40	0.71	0.39	1.30
**Substance use**												
None	1			1			1			1		
Cigarettes	**1.54**	**1.28**	**1.86**	1.61	**1.31**	**1.98**	0.84	0.33	2.14	0.81	0.30	2.18
Alcohol	**1.31**	**0.92**	**1.86**	1.36	0.94	1.95	1.18	0.28	4.94	1.42	0.33	6.15
Betel quid	**2.62**	**1.19**	**5.80**	2.21	0.99	4.94	-	-	-	-	-	-
Cigarettes+Alcohol	**1.55**	**1.20**	**1.99**	1.60	**1.23**	**2.10**	1.66	0.65	4.24	1.67	0.62	4.54
Cigarettes+Betel quid	**1.68**	**1.11**	**2.53**	1.71	**1.11**	**2.62**	1.03	0.14	7.54	1.17	0.15	8.97
Alcohol+Betel quid	1.71	0.71	4.12	1.67	0.68	4.08	4.91	0.65	36.88	6.54	0.83	51.46
Cigarettes+Alcohol+Betel quid	**1.92**	**1.49**	**2.47**	2.00	**1.52**	**2.63**	1.59	0.56	4.48	1.83	0.60	5.52
*p* value for trend				< 0.0001						0.1616		

Abbreviations: cOR, crude odds ratio; aOR, adjusted odds ratio; CI, confidence interval; GU, gastric ulcer.

^1^Use stomach normal as reference category. Adjusted for all variables listed in this table.

Bold indicates statistical significance.

**Table 5:** Risk factors for developing duodenum disease in 9,275 patients

	Duodenitis						DU					
	cOR^1^	95%CI		aOR^1^	95%CI		cOR^1^	95%CI		aOR^1^	95%CI	
**Gender**												
Female	**1**			**1**			**1**			**1**		
Male	**2.13**	**1.80**	**2.53**	**1.65**	**1.35**	**2.01**	**2.05**	**1.83**	**2.28**	**1.71**	**1.51**	**1.94**
**Age**												
20–49	1			1			1			1		
50–69	**0.74**	**0.62**	**0.88**	**0.72**	**0.60**	**0.86**	**1.41**	**1.25**	**1.59**	**1.42**	**1.26**	**1.61**
70–97	0.88	0.69	1.13	0.90	0.70	1.16	**1.63**	**1.39**	**1.92**	**1.62**	**1.37**	**1.91**
**BMI**												
< 18.5, underweight	0.74	0.51	1.09	0.77	0.52	1.14	0.65	0.51	0.83	0.71	0.56	0.91
18.5–24.9, normal	1			1			1			1		
> 25, overweight	**1.50**	**1.27**	**1.78**	**1.38**	**1.16**	**1.63**	**1.14**	**1.02**	**1.27**	1.03	0.92	1.16
**Substance use**												
None	1			1			1			1		
Cigarettes	**1.84**	**1.44**	**2.33**	**1.38**	**1.07**	**1.79**	**1.82**	**1.55**	**2.12**	**1.45**	**1.23**	**1.71**
Alcohol	1.34	0.86	2.11	1.04	0.65	1.64	1.05	0.76	1.43	0.83	0.60	1.15
Betel quid	1.47	0.45	4.84	1.27	0.38	4.22	0.85	0.33	2.21	0.66	0.26	1.73
Cigarettes+Alcohol	**1.72**	**1.23**	**2.41**	1.27	0.89	1.81	2.32	1.90	2.83	**1.80**	**1.46**	**2.22**
Cigarettes+Betel quid	**3.14**	**2.01**	**4.90**	**2.17**	**1.37**	**3.44**	**1.89**	**1.32**	**2.70**	1.37	0.95	1.98
Alcohol+Betel quid	**2.73**	**1.04**	**7.14**	1.97	0.75	5.21	1.33	0.58	3.08	1.00	0.43	2.32
Cigarettes+Alcohol+Betel quid	**3.02**	**2.27**	**4.03**	**2.12**	**1.55**	**2.89**	**1.75**	**1.40**	**2.21**	**1.29**	**1.01**	**1.65**
*p* value for trend				< 0.0001						< 0.0001		

Abbreviations: cOR, crude odds ratio; aOR, adjusted odds ratio; CI, confidence interval; DU, duodenal ulcer.

^1^Use duodenum normal as reference category. Adjusted for all variables listed in this table.

Bold indicates statistical significance.

Compared with non-smokers, people who smoked were 1.27 times more likely (95% CI: 1.09–1.47), people who smoked and drank were 1.38 times more likely (95% CI: 1.13–1.68), and those who used all three substances habitually were 1.60 times more likely (95% CI: 1.29–2.00) to be diagnosed with grade A-B reflux esophagitis (Table 3). Alcohol drinkers were 5.56 times, 6.73 times, and 12.35 times more likely to be diagnosed with esophageal cancer whether they drank alcohol only or in combination one other substance (cigarette smoking or betel quid). However, a person who smoke, drank and chewed habitually simultaneously was 17.28 times more likely (95% CI: 7.59–39.33) to be diagnosed with esophageal cancer and 2.99 times more likely (95% CI: 2.04–4.39) to be diagnosed as having Barrette's esophagus (Table 3). People who smoked only, who also drank and/or chewed were 1.61 (95% CI: 1.31–1.98), 1.60 (95% CI: 1.23–2.10), 1.71 (95% CI: 1.11–2.62) and 2.00 (95% CI: 1.52–2.63) times more likely to be diagnosed with gastric ulcers, respectively (Table 4). The risk of duodenitis and duodenal ulcer was also significantly increased among smokers (aOR: 1.38 and 1.45, respectively) (Table 5). There was a significant trend of higher risk for all upper digestive tract diseases except gastric cancer if a subject used more substances in combination.

Information on history of diabetes mellitus, hypertension and family history of cancer were available beginning September 2010. We performed a subgroup analysis (N = 4,341, Supplementary Table 2) and found those three factors did not influence the effect of substance use (Supplementary Tables 3–5). Moreover, people who had hypertension were 1.35 times more likely (95% CI: 1.08–1.68) to have gastric ulcer (Supplementary Table 4). Those with diabetes was 1.37 times more likely (95% CI: 1.09–1.73) to be diagnosed of duodenal ulcer (Supplementary Table 5).

### Meta-analysis

**Figure 2:**
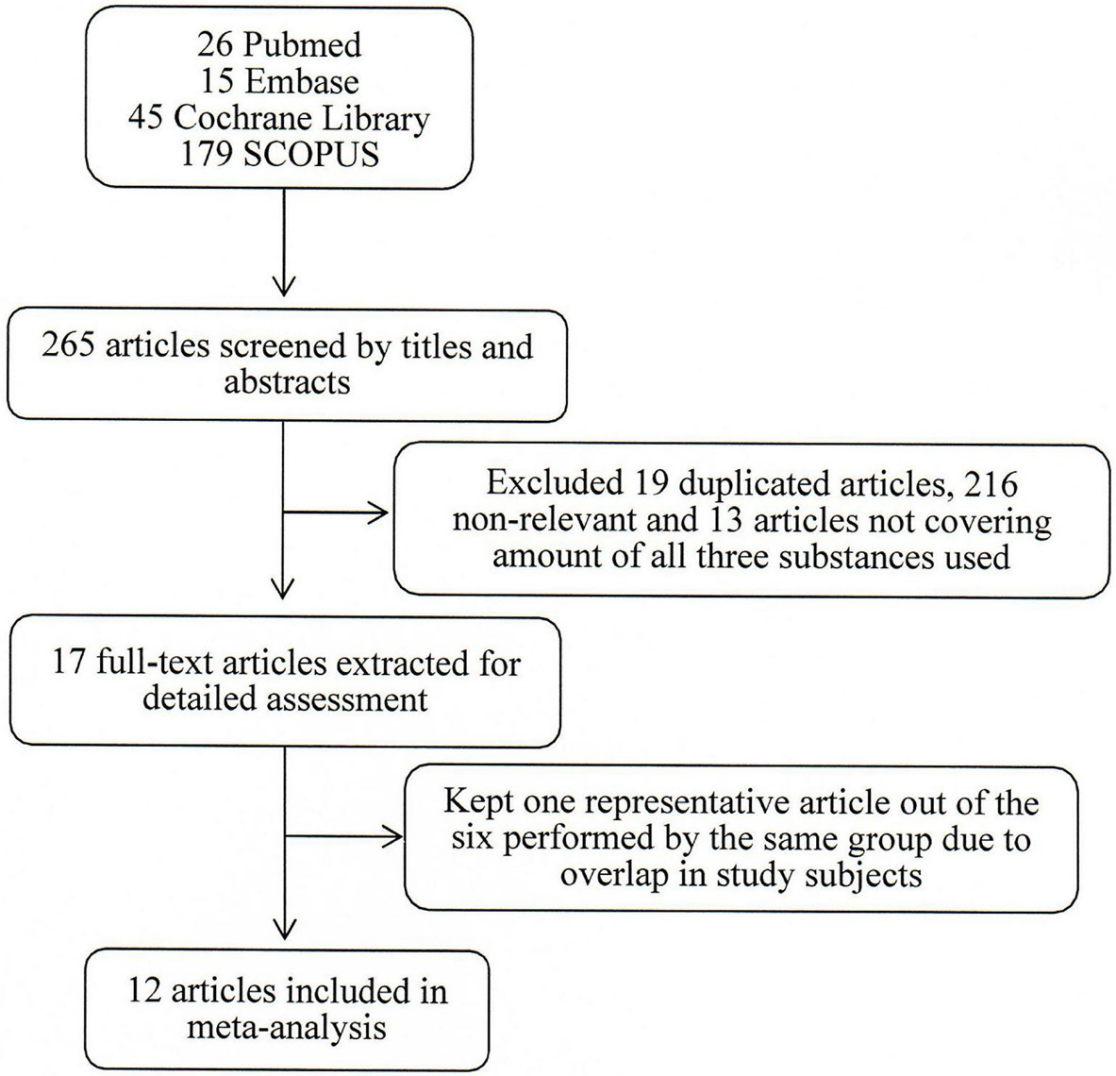
Screening and selection of previous studies investigating effect of substances use on esophageal disease

The initial search found 265 epidemiological studies in our search of the literature (Figure 2). We excluded 19 duplicated articles, 216 non-relevant articles, and 13 articles that either did not cover all three substances or the data they provided were not provided in table form. Six articles were published by the same research group and the study subjects were overlapping, so we only included the most representative one. Finally, we included 12 studies in our meta-analysis [18–20, 22–30].

**Figure 3:**
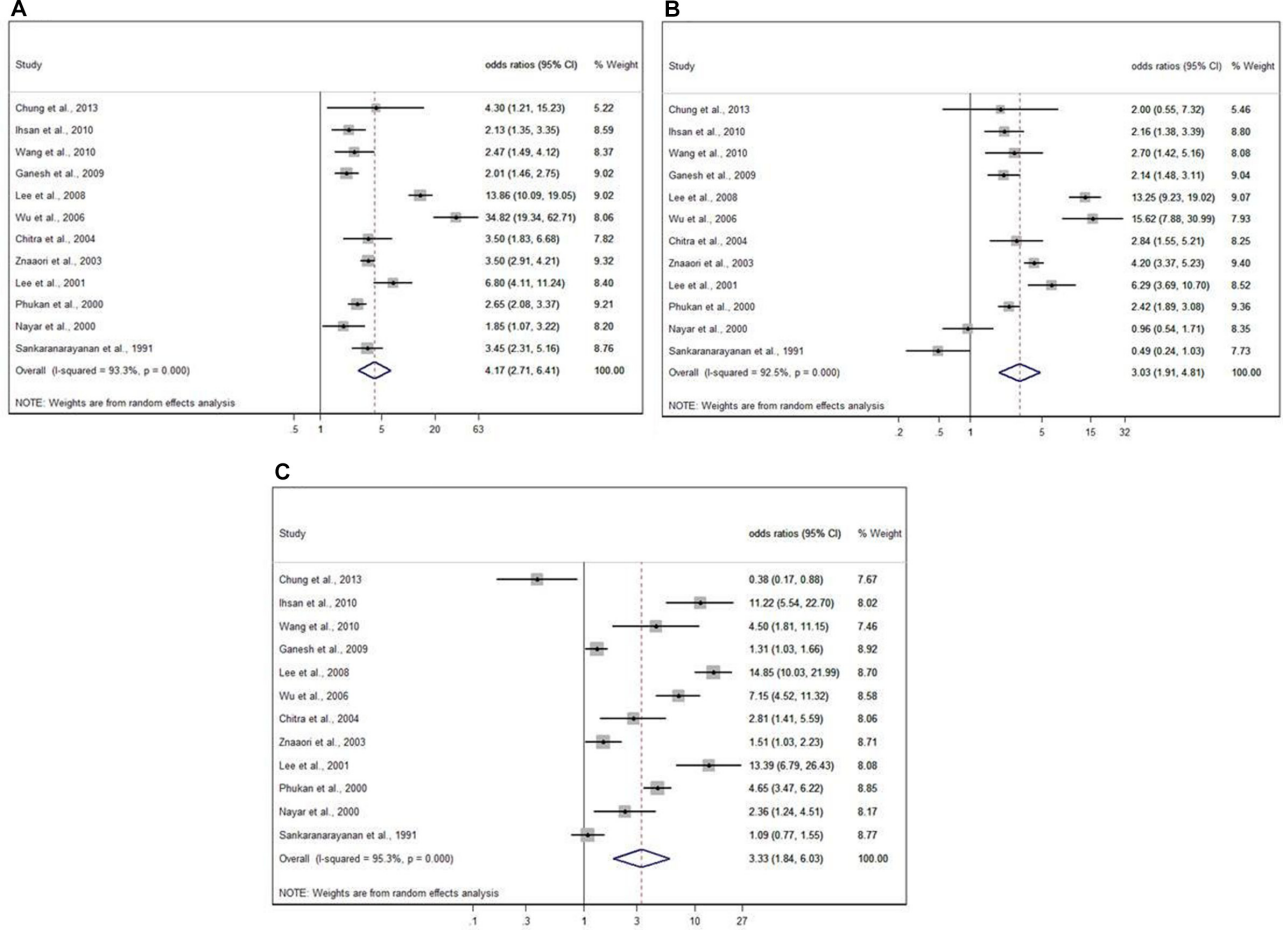
Meta-analysis by random effect model for substance use and esophageal disease (**A**) Odds ratios (95% CI) for esophageal disease in alcohol drinkers versus non-drinkers in previous studies. (**B**) Odds ratio (95% CI) for esophageal disease in cigarette smokers versus non-smokers in previous studies. (**C**) Odds ratio (95% CI) for esophageal disease in betel quid chewers versus non-chewers in previous studies.

Only one study focused on reflux esophagitis [26] and the rest focused on esophageal cancer. We found habitual use of alcohol to carry a significantly higher risk of any esophageal disease (odds ratio [OR] = 4.17; 95% CI = 2.71–6.41) in the random effects model (heterogeneity test, I^2^ = 93.3%; *p* < 0.001) (Figure 3A). Similar higher risks of any esophageal disease among smokers and betel nut chewers were also found for smokers (OR = 3.03 and 95% CI: 1.91–4.81) and betel nut chewers (OR = 3.33 and 95% CI: 1.84–6.03), although the heterogeneity tests were relatively high for both (I^2^ = 92.5% and *p* < 0.001 for smokers and 95.3% and *p* < 0.001 for betel nut chewers) (Figure 3B and 3C).

## DISCUSSION

In this hospital-based survey, the incidence of esophageal cancer and gastric adenocarcinoma was 0.55% and 0.60%, respectively. Smoking was an important risk factor for upper digestive diseases. Because we only had twelve participants with grade C-D reflux esophagitis, it was difficult to study the risk factors for this disease. Alcohol drinking was also associated with the risk of esophageal cancer. It added to the adverse effect of smoking on reflux esophagitis, Barrett's esophagus, gastric ulcer, duodenal ulcer and esophageal cancer. Patients who habitually used cigarettes, alcohol, and betel nut simultaneously were at significantly greater risk of esophageal cancer, Barrett's esophagus, reflux esophagitis, gastric ulcer, duodenal ulcer, and gastric cancer.

One Danish study found smoking but not drinking to be a main risk factor for peptic ulcer disease [31]. A meta-analysis also reported smoking to be an important risk factor for peptic ulcers [32]. Smoking is a well-established risk factor for gastric ulcer. The current study found that patients who smoked only had 1.6 times the risk for gastric ulcer than patients who did not smoke, a finding similar to those of previous studies [5, 32]. Like the current study, one cross-sectional study in Japan did not find an association between alcohol and either gastric ulcer or duodenal ulcer [33]. However, the current study found that alcohol drinking and betel quid chewing added to smoking's effect on gastric ulcer and that alcohol also added to smoking's effect on duodenal ulcer.

Two large cohort studies and one meta-analysis have reported smoking to be a risk factor for gastric cancer [14, 15, 34]. Another meta-analysis found that, except in cases of very heavy drinking, alcohol was not strongly associated with gastric cancer [35]. Few studies have discussed the relationship between betel quid chewing and gastric cancer and the results were inconsistent [21, 36]. Our previous case-control study found betel quid to be a significant risk factor for gastric cancer [21]. However, another study in India indicated an inverse association between them [36]. This discrepancy might arise from certain gene-environmental interactions. Moreover, the components of betel quid used in Taiwan are different from those used in India where the quid often contain tobacco. We found no significant association between smoking, alcohol, betel nut and gastric cancer in this study. This lack of significance may be due to small number of cases in each group. Moreover, we did not have enough data on the status of Helicobacter pylori, an important cause of chronic inflammation, atrophy and cancer of stomach [37].

Previous studies have reported an association between gender and gastro-esophageal reflux disease and Barrett's esophagus [38, 39]. In this study, we also found a positive trend for these diseases in our male subjects. One possible explanation for these findings may be that women may be more aware of the different symptoms and be more likely to seek treatment in mild stages or that women in Taiwan have lower rates of habitual use of cigarette smoking, alcohol drinking and betel quid chewing. Our results also showed that increasing age and higher BMI were risk factors for the development of gastro-esophageal reflux disease and Barrett's esophagus. These findings were consistent with the previous studies [40–42] and further demonstrate the validity of this study.

One cross-sectional study in Japan reported smoking to be associated with reflux esophagitis and Barrett's esophagus and excess alcohol consumption to be a risk factor for Barrett's esophagus while the frequency of alcohol consumption not to be a risk factor for either reflux esophagitis or Barrett's esophagus [11]. A case-control study in Italy also reported smoking be a risk factor for Barrett's esophagus and reflux esophagitis [43]. One review of 46 studies showed the risk factors for Barrett's esophagus to be male gender, older age, higher BMI and smoking [44]. Only two of the studies reviewed in that article showed excessive alcohol consumption to be strongly associated with Barrett's esophagus [44]. It is still controversial whether there is a relationship between alcohol drinking and Barrett's esophagus. In our study, smoking, male gender and BMI > 25 were risk factors for reflux esophagitis and Barrett's esophagus. Although we did not find that alcohol drinking only or betel quid chewing only contributed to the development of reflux esophagitis and Barrett’ esophagus, we did find that they added to smoking's effect on the two diseases.

Freedom et al. reported an association between smoking and alcohol drinking and esophageal squamous cell carcinoma [14]. In China, where there are more than 100,000 new cases of esophageal cancer per year, smoking and alcohol drinking are risk factors in esophageal cancer [45]. Zeka et al. performed a meta-analysis study finding combined use of tobacco and alcohol to be a risk factor for esophageal cancer [46].Our previous studies found betel nut use to be a risk factor of esophageal cancer [17, 19, 20]. In the current study, we found that the simultaneous habitual use of cigarette, alcohol and betel nut had a synergistic effect on the development of this cancer.

The mechanisms through which consumption of cigarette, alcohol and, betel quid may lead to upper digestive diseases are complicated. Smoking can cause chronic inflammation and carcinogenesis through different mechanisms, including alteration of mucosal cell proliferation, change of blood flow at the inflammatory sites, resulting in tumor cell proliferation and angiogenesis in gastrointestinal tract [47]. In one rat model, ethanol-induced gastritis was found to progress into gastric ulcer as a result of apoptosis in the gastric mucosa stimulated by TNF-α overexpression [48]. Ethanol results in cytotoxic activity in mucosa, increases stem cell division and promotes malignant transformation [49]. Different alkaloids including guvacine, arecoline, arecaidine and guvacoline are the components of areca nut that can induce systemic effects and arecoline can cause carcinoma [50]. Gastric acid hypersecrection is one of the etiologies of reflux esophagitis and peptic ulcer disease. In another study using rats, betel quid chewing was found to increase the release of histamine and gastric acid [51].

In our subgroup analysis, we found diabetes and hypertension to be significantly associated with peptic ulcer diseases but not esophageal or gastric cancers, which was consistent with previous studies [52, 53]. In the diabetic rats, gastric ulcer healing is significant delayed due to release of proinflammatory cytokines and the attenuation of angiogenesis [54]. Moreover, diabetic or hypertensive patients have higher chance to develop cardiovascular diseases or other comorbidities, and medication prescribed to control them, such as aspirin and nonsteroidal anti-inflammatory drugs, can influence mucosal integrity to induce ulcer [55]. Although we did not have complete data to adjust for medication history, we did not find the effects of cigarette, alcohol and betel quid consumption on upper gastrointestinal diseases to be influenced by diabetes or hypertension history.

This study has several limitations. First, we did not include Helicobacter pylori, an important cause of gastric cancer and peptic ulcer. Nor did we have the information on drug usage for aspirin and nonsteroidal anti-inflammatory drugs, which are potential risk factors for peptic ulcer. Second, this study is a hospital-based study and most patients received endoscopies because they had gastrointestinal symptoms, which would lead to a higher prevalence of upper digestive disease than would be found in the general population. Third, we did not have biochemical data for every patient, because this study was conducted in an out-patient setting. Fourth, the information on substance use was collected by an interview and recall bias was likely to happen. However, we have confirmed the high reliability of the questionnaire in the supportive study in the methods. Finally, the meta-analysis was confined to esophageal diseases, especially esophageal cancer because few studies covered all three substances and other upper digestive diseases.

In conclusion, this study found cigarette smoking, alcohol drinking and betel quid chewing to be independently associated with upper digestive tract disease. People who use all three substances habitually are at significantly higher risk of Barrett's esophagus and esophageal cancer. Alcohol drinking and betel quid chewing add to smoking's effect on reflux esophagitis and gastric ulcers.

## MATERIALS AND METHODS

### Study subjects

This study enrolled participants who filled out questionnaires and received upper endoscopies offered at the outpatient clinics at one medical center, KMUH, and three regional hospitals in southern Taiwan-KMHK, PT and HC from April 2008 to December 2013. We recruited 10,611 participants aged 20 years and over from the four hospitals. We excluded participants who did not have complete endoscopic reports or those who had been previously diagnosed and treated for gastric-esophagus cancers or who had previously received upper digestive tract operations. This study was approved by the ethics committee of Kaohsiung Medical University Hospital (KMUH-IRB-990241). All participants provided written informed consent. All clinical investigations were conducted in accordance with the principles expressed in the declaration of Helsinki.

### Questionnaire

Each participant was interviewed by a trained interviewer using a standardized questionnaire collecting demographic information (age, gender, height, weight and circumference of waist and hip) and history of substance use (alcohol, cigarette and betel quid). Dietary habits such as consumption of vegetables, fruit, red meat, tea, coffee and hot beverages were also recorded. Substance use was defined in a participant if he or she were consuming or had consumed any alcoholic beverage at least one time per week, smoked or had smoked ten cigarettes or more per week and or chewed or had chewed one betel quid or more per day for at least one year. Past history of diabetes mellitus, hypertension, family history of cancer and Chinese GERD Q questionnaire were added into the questionnaire starting from September 2010.

### Reliability of three substance use in questionnaire

Our previous study found the questionnaire-collected data on history of substance use (alcohol, cigarette and betel quid) to be reliable, based on comparisons of answers of questionnaires with measurements of safrole-DNA adducts, urinary cotinine and creatinine and serum acetaldehyde, all known markers of substance use [56]. We also recruited participants from Department of Gastroenterology and Department of Physical Medicine and Rehabilitation in KMHK to evaluate test-retest reliability (Supplementary Table 6 and Supplementary Figure 1). A trained interviewer randomly selected participants from these two departments to take part in a telephone interview one month after their first questionnaire. We then compared the results from Department of Gastroenterology (*n* = 207) and Department of Physical Medicine and Rehabilitation (*n* = 202) and analyzed categorical and continuous data using Kappa coefficient statistic and Spearman's correlation coefficient, respectively. The Kappa coefficient was more than 0.7 and Spearman's correlation coefficient more than 0.8, indicating acceptable reliability. This supportive study was approved by the ethics committee of Kaohsiung Medical University Hospital (KMUH-IRB-960204).

### Confirmatory clinical diagnosis

All patients received endoscopies using Olympus video-endoscopes (Olympus Corporation, Tokyo, Japan) and the medical diagnoses of endoscopic records were re-confirmed by gastroenterologists (MC Wu and YK Wang). Biopsy specimens from sites suspected of malignancy or Barrett's esophagus were also collected and all cancer diagnoses were histologically confirmed by two different pathologists.

### Statistical analysis

Categorical variables were summarized as count (%) and the comparisons between risk factors and clinical disease category analyzed using Chi-square test. Polynomial regressions were used to analyze the association between risk factors and participants’ diseases of the esophagus, stomach and duodenum.

Because 6.63% and 0.50% of the questionnaires had missing BMI data and missing substance use information, we performed multiple imputation to handle missing data following Monotone Logistic Regression Method [57]. To do this, missing data were recreated by imputing value from five duplicate values. We used the imputed dataset in our analyses (Table 1) and also showed the distribution of raw data in Supplementary Table 2. A two-tailed *p* value < 0.05 was considered significant. All statistical operations were performed using SAS 9.4 statistical software [58].

### Meta-analysis

A meta-analysis following the PRISMA checklist was conducted to examine the association between habitual use of these substances and esophageal diseases. To do this, we searched the databases PubMed, Embase, Cochrane Library and SCOPUS from January 01, 1970 to July 31, 2016. The search terms were ((“oesophageal disease”[All Fields] OR “esophageal diseases”[MeSH Terms] OR (“esophageal”[All Fields] AND “diseases”[All Fields]) OR “esophageal diseases”[All Fields] OR (“esophageal”[All Fields] AND “disease”[All Fields]) OR “esophageal disease”[All Fields]) AND (“smoking”[MeSH Terms] OR “smoking”[All Fields]) AND (“alcohol drinking”[MeSH Terms] OR (“alcohol”[All Fields] AND “drinking”[All Fields]) OR “alcohol drinking”[All Fields]) AND (“areca”[MeSH Terms] OR “areca”[All Fields] OR (“areca”[All Fields] AND “nut”[All Fields]) OR “areca nut”[All Fields])).

These studies were first screened from titles and abstracts then by reviewing their full-texts. A study was included if its alcohol, cigarette and betel quid samples were characterized in tables. Combined odds ratio and 95% CI were used to measure the association between alcohol drinking, cigarette smoking and betel quid chewing, respectively. Heterogeneity across studies was evaluated by the Q test and I^2^ statistic. Random-effect model was used for this meta-analysis. All statistical operations were performed using STATA 14.0 software.

## SUPPLEMENTARY MATERIALS FIGURES AND TABLES





## Abbreviations

GERD: gastroesophageal reflux disease
EV: esophageal varices
GIST: Gastrointestinal Stromal Tumor
MALToma: Mucosa-associated lymphoid tissue *lymphoma*;
GV: gastric varices
OR: odds ratio
cOR: crude odds ratio
aOR: adjusted odds ratio
CI: confidence interval
