# Supplementary material for: Effects of alcohol consumption, cigarette smoking, and betel quid chewing on upper digestive diseases: a large cross-sectional study and meta-analysis
Source: Oncotarget. 2017 Sep 11;8(44):78011–22. doi: 10.18632/oncotarget.20831 (PMC5652832; doi:10.18632/oncotarget.20831)
Supplement: Supplementary file 1 [file oncotarget-08-78011-s001.pdf]

Effects of alcohol consumption, cigarette smoking, and betel quid chewing on upper digestive diseases: a large cross-sectional study and meta-analysis

SUPPLEMENTARY MATERIALS

Supplementary Table 1: Baseline characteristics of participants receiving gastrointestinal endoscopic examination at different hospitals

	KMUH (*N* = 4896)		KMHK (*N* = 3541)		PT (*N* = 838)	
	N	%	*N*	%	*N*	%
**Gender**	N					
Female	2435	49.73	1761	49.73	514	61.34
Male	2461	50.27	1780	50.27	324	38.66
**Age**						
20-29	392	8.01	294	8.3	26	3.1
30-49	1334	27.25	1252	35.36	144	17.18
50-69	2421	49.45	1586	44.79	480	57.28
70-97	749	15.3	409	11.55	188	22.43
**BMI**						
< 18.5, underweight	373	7.62	199	5.62	64	7.64
18.5-24.9, normal	2624	53.59	2036	57.5	485	57.88
> 25, overweight	1899	38.79	1306	36.88	289	34.49
**Substance use**						
None	3400	69.44	2525	71.31	676	80.67
Cigarettes	610	12.46	417	11.78	59	7.04
Alcohol	138	2.82	135	3.81	30	3.58
Betel quid	18	0.37	12	0.34	7	0.84
Cigarettes +Alcohol	299	6.11	219	6.18	33	3.94
Cigarettes +Betel quid	118	2.41	55	1.55	8	0.95
Alcohol+Betel quid	20	0.41	15	0.42	3	0.36
Cigarettes+Alcohol+Betel quid	293	5.98	163	4.6	22	2.63

Abbreviations: KMUH, Kaohsiung Medical University Hospital; KMHK, Kaohsiung Municipal Hsiao-Kang Hospital; PT, Ministry of Health and Welfare Ping-Tung Hospital.

Supplementary Table 2: Histopathological characteristics of the subgroup 4,357 patients with complete past history and cancer family history

	Number	%
**Esophagus**		
Normal	2425	55.66
GERD A-B	1564	35.90
GERD C-D	8	0.18
Barrett's esophagus	214	4.91
Esophageal cancer	29	0.67
Other (ulcer, Mallory-Weiss tear, EV and papilloma)	117	2.69
**Stomach**		
Normal	3781	86.78
GU	453	10.40
Gastric polyp	2	0.05
Gastric adenocarcinoma	24	0.55
GIST, lymphoma, MALToma	53	1.22
Other(GV and other cancer)	44	1.01
**Duodenum**		
Normal	3316	76.11
Duodenitis	264	6.06
DU	775	17.79
Tumor	2	0.05

Abbreviations: GERD, gastroesophageal reflux disease; EV, esophageal varices; GU, gastric ulcer; GIST, Gastrointestinal Stromal Tumor; MALToma, Mucosa-associated lymphoid tissue *lymphoma;* GV, gastric varices; DU, duodenal ulcer.

Supplementary Table 3: Subgroup analysis of risk factors for developing esophagus disease (*N* = 4,357). See Supplementary_Table_3

Supplementary Table 4: Subgroup analysis of risk factors for developing stomach disease (*N* = 4,357)

	GU						Gastric adenocarcinoma					
	**cOR1**	**95%CI**		**aOR1**	**95%CI**		**cOR1**	**95%CI**		**aOR1**	**95%CI**	
**Gender**												
Female	1			1			1			1		
Male	1.53	1.25	1.86	1.16	0.93	1.46	0.66	0.29	1.52	0.78	0.31	1.98
**Age**												
20-49	1			1			1			1		
50-69	2.43	1.87	3.17	**2.25**	**1.71**	**2.96**	2.55	0.71	9.15	2.87	0.78	10.55
70-97	3.66	2.69	4.98	**3.21**	**2.30**	**4.47**	8.35	2.29	30.44	**8.27**	**2.08**	**32.85**
**BMI**												
< 18.5, underweight	0.73	0.46	1.16	0.83	0.52	1.33	2.89	1.12	7.44	**2.74**	**1.04**	**7.19**
18.5-24.9, normal	1			1			1			1		
> 25, overweight	1.44	1.18	1.76	**1.30**	**1.06**	**1.60**	0.19	0.04	0.82	**0.19**	**0.04**	**0.85**
**Substance use**												
None	1			1			1			1		
Cigarettes	1.46	1.09	1.97	**1.47**	**1.06**	**2.02**	0.34	0.05	2.51	0.45	0.06	3.58
Alcohol	1.42	0.86	2.33	1.42	0.85	2.38	-	-	-	-	-	-
Betel quid	3.21	1.16	8.90	**2.90**	**1.02**	**8.20**	-	-	-	-	-	-
Cigarettes +Alcohol	1.82	1.25	2.65	**1.90**	**1.27**	**2.85**	0.70	0.09	5.21	1.07	0.13	8.70
Cigarettes +Betel quid	1.41	0.74	2.69	1.33	0.68	2.59	-	-	-	-	-	-
Alcohol+Betel quid	1.38	0.41	4.64	1.20	0.35	4.14	-	-	-	-	-	-
Cigarettes +Alcohol+Betel quid	1.76	1.16	2.66	**1.67**	**1.07**	**2.60**	0.84	0.11	6.26	1.45	0.17	12.14
**Diabetes**												
No	1			1			1			1		
Yes	1.82	1.42	2.35	1.23	0.95	1.61	1.07	0.32	3.59	0.91	0.26	3.20
**Hypertension**												
No	1			1			1			1		
Yes	1.90	1.55	2.33	**1.35**	**1.08**	**1.68**	1.47	0.63	3.44	1.12	0.44	2.83
**Cancer family cancer**												
No	1			1			1			1		
Yes	0.71	0.55	0.90	0.73	0.57	0.93	0.94	0.37	2.36	1.03	0.40	2.64

Abbreviations: cOR, crude odds ratio; aOR, adjusted odds ratio; CI, confidence interval.
[1]Using stomach normal as reference category. Adjusted odds ratio were adjusted for all variables listed in this table.
Bold indicates statistical significance.

Supplementary Table 5: Subgroup analysis of risk factors for developing duodenum disease (N = 4,357)

	Duodenitis						DU					
	cOR[1]	95%CI		aOR[1]	95%CI		cOR[1]	95%CI		aOR[1]	95%CI	
**Gender**												
Female	1			1			1			1		
Male	2.13	1.65	2.77	**1.77**	**1.32**	**2.37**	1.81	1.54	2.12	**1.57**	**1.31**	**1.88**
**Age**												
20-49	0.74	0.56	0.98	**0.73**	**0.54**	**0.97**	1.34	1.12	1.61	**1.37**	**1.13**	**1.66**
50-69	1			1			1			1		
70-97	1.07	0.74	1.54	1.10	0.74	1.63	1.72	1.36	2.18	**1.75**	**1.35**	**2.26**
**BMI**												
<18.5, underweight	0.69	0.38	1.27	0.72	0.39	1.33	0.75	0.54	1.06	0.80	0.56	1.12
18.5-24.9, normal	1			1			1			1		
>25, overweight	1.50	1.16	1.94	**1.43**	**1.09**	**1.86**	1.13	0.96	1.34	1.09	0.92	1.28
**Substance use**												
None	1			1			1			1		
Cigarettes	1.28	0.84	1.94	0.94	0.61	1.46	1.66	1.31	2.10	**1.36**	**1.06**	**1.75**
Alcohol	1.58	0.85	2.92	1.20	0.64	2.26	1.12	0.72	1.73	0.94	0.60	1.46
Betel quid	0.79	0.11	5.92	0.66	0.09	5.02	0.26	0.03	1.93	0.21	0.03	1.60
Cigarettes +Alcohol	1.85	1.10	3.09	1.35	0.79	2.30	2.25	1.66	3.04	**1.84**	**1.34**	**2.54**
Cigarettes +Betel quid	1.82	0.86	3.84	1.22	0.56	2.63	1.04	0.58	1.86	0.75	0.41	1.36
Alcohol+Betel quid	0.75	0.10	5.61	0.57	0.08	4.34	0.73	0.22	2.48	0.61	0.18	2.07
Cigarettes +Alcohol+Betel quid	3.53	2.29	5.45	**2.41**	**1.51**	**3.85**	1.67	1.16	2.40	1.29	0.89	1.89
**Diabetes**												
No	1			1			1			1		
Yes	1.45	1.02	2.05	1.37	0.95	1.98	1.54	1.24	1.92	**1.37**	**1.09**	**1.73**
**Hypertension**												
No	1			1			1			1		
Yes	0.97	0.73	1.29	0.83	0.61	1.13	1.04	0.87	1.23	0.83	0.68	1.00
**Cancer family history**												
No	1			1			1			1		
Yes	0.82	0.61	1.11	0.89	0.66	1.21	0.91	0.76	1.09	0.95	0.79	1.14

Abbreviations: cOR, crude odds ratio; aOR, adjusted odds ratio; CI, confidence interval

[1]Using duodenum normal as reference category. Adjusted odds ratio were adjusted for all variables listed in this table.

Bold indicates statistical significance.

Supplementary Table 6: Comparison of results of first and second interviews from department of gastroenterology and department of physical medicine and rehabilitation

	Department of Gastroenterology (*n* = 207)				Department of Physical Medicine and Rehabilitation (*n* = 202)			
	Second interview				Second interview			
	**Current**	**Former**	**Never**	**Kappa**	**Current**	**Former**	**Never**	**Kappa**
First interview								
Cigarettes								
Current	40	0	0	0.9867	53	0	1	
Former	1	6	0		0	7	1	0.9665
Never	0	0	160		1	0	139	
Alcohol								
Current	16	0	5	0.8289	11	0	2	0.7991
Former	0	3	2		0	2	2	
Never	0	0	181		2	0	183	
Betel nut								
Current	2	0	0	0.9072	13	0	3	0.7952
Former	0	3	1		0	2	3	
Never	0	0	201		1	0	180	

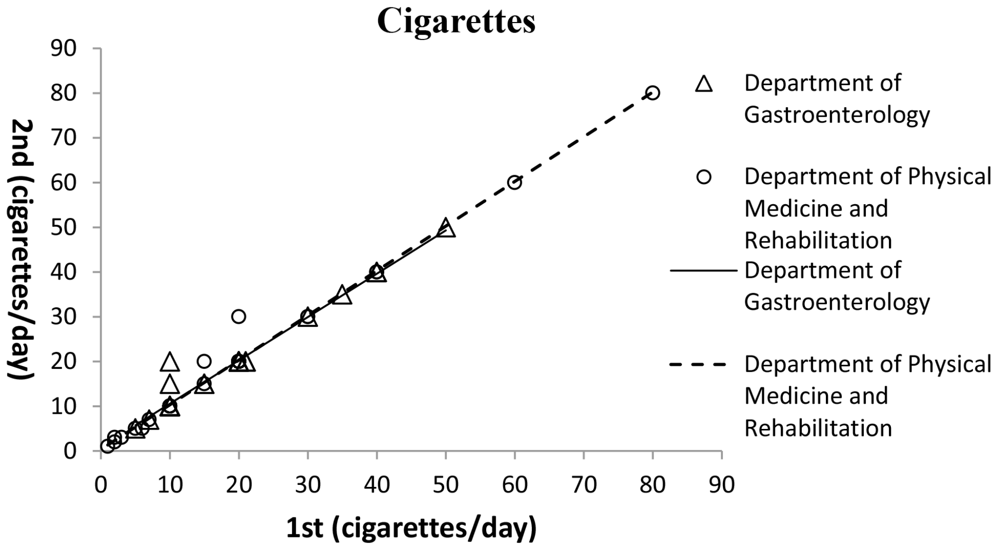

Spearman correlation
Department of Gastroenterology : R = 0.9536, $p < 0.0001$, $N = 43$
Department of Physical Medicine and Rehabilitation : R = 0.9815, $p < 0.0001$, $N = 48$

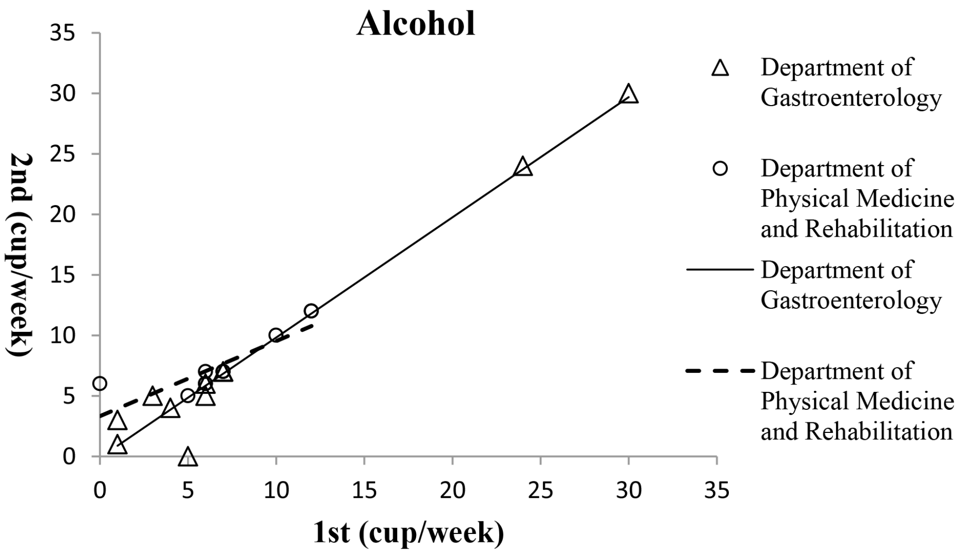

Spearman correlation
Department of Gastroenterology : R = 0.9073, $p <0.0001$, $N = 12$
Department of Physical Medicine and Rehabilitation : R = 0.9328, $p < 0.0001$, $N = 11$

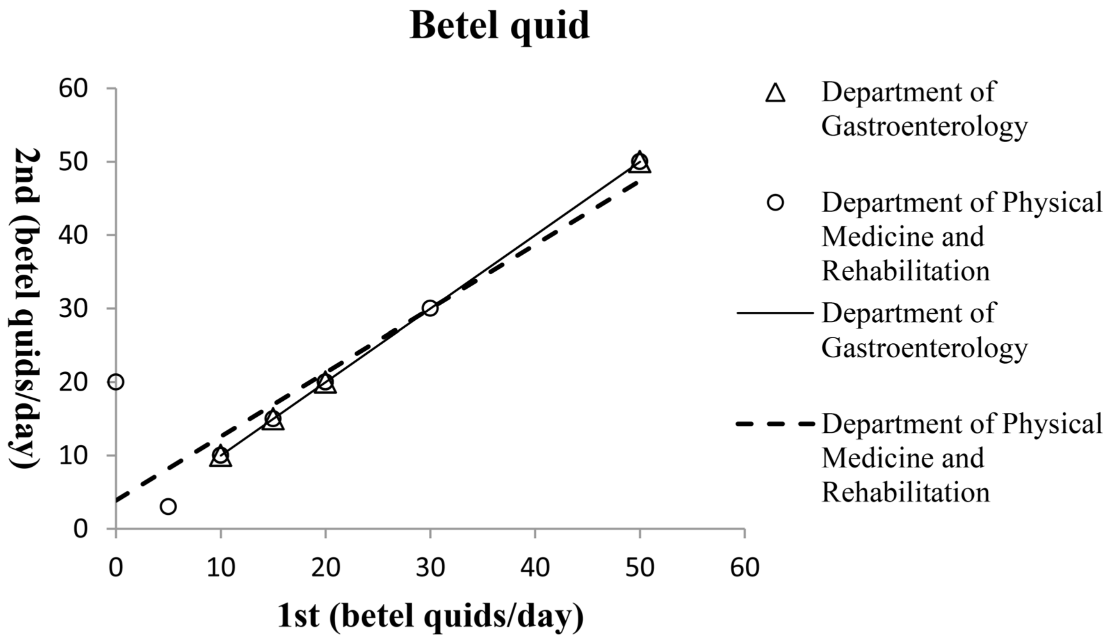

Spearman correlation
Department of Gastroenterology : R = 1.0000, $p < 0.0001$, $N = 5$
Department of Physical Medicine and Rehabilitation : R = 0.8522, $p < 0.0001$, $N = 14$

Supplementary Figure 1: Spearman's correlations of three substances from first and second interviews from Department of Gastroenterology and Department of Physical Medicine and Rehabilitation
