# Supplementary material for: Effects of alcohol consumption, cigarette smoking, and betel quid chewing on upper digestive diseases: a large cross-sectional study and meta-analysis
Source: Oncotarget. 2017 Sep 11;8(44):78011–22. doi: 10.18632/oncotarget.20831 (PMC5652832; doi:10.18632/oncotarget.20831)
Supplement: Supplementary file 2 [file oncotarget-08-78011-s002.docx]

Supplementary Table 3: Subgroup analysis of risk factors for developing esophagus disease (N= 4,357)

|  | | **GERD A-B** | | | | | | **GERD C-D** | | | | | | **Barrett's esophagus** | | | | | | | **Esophageal cancer** | | | | | | | | |
| --- | --- | --- | --- | --- | --- | --- | --- | --- | --- | --- | --- | --- | --- | --- | --- | --- | --- | --- | --- | --- | --- | --- | --- | --- | --- | --- | --- | --- | --- |
|  | | **cOR^1^** | **95%CI** | | **aOR^1^** | **95%CI** | | **cOR^1^** | **95%CI** | | **aOR^1^** | **95%CI** | | **cOR^1^** | | **95%CI** | | **aOR^1^** | **95%CI** | | **cOR^1^** | | **95%CI** | | | | **aOR^1^** | **95%CI** | |
| **Gender** | |  |  |  |  |  |  |  |  |  |  |  |  |  | |  |  |  |  |  |  | |  | |  | |  |  |  |
| Female | | 1 |  |  | 1 |  |  | 1 |  |  | 1 |  |  | 1 | |  |  | 1 |  |  | 1 | |  | |  | | 1 |  |  |
| Male | | 1.94 | 1.70 | 2.20 | **1.52** | **1.31** | **1.76** | 1.49 | 0.37 | 5.98 | 0.98 | 0.20 | 4.71 | 3.27 | | 2.43 | 4.42 | **1.99** | **1.42** | **2.81** | 20.07 | | 4.77 | | 84.38 | | **6.97** | **1.42** | **34.15** |
| **Age** | |  |  |  |  |  |  |  |  |  |  |  |  |  | |  |  |  |  |  |  | |  | |  | |  |  |  |
| 20-49 | | 1 |  |  | 1 |  |  | 1 |  |  | 1 |  |  | 1 | |  |  | 1 |  |  | 1 | |  | |  | | 1 |  |  |
| 50-69 | | 1.13 | 0.98 | 1.30 | 1.12 | 0.96 | 1.30 | 0.45 | 0.08 | 2.69 | 0.54 | 0.09 | 3.36 | 1.10 | | 0.80 | 1.52 | 1.09 | 0.78 | 1.53 | 3.69 | | 1.27 | | 10.76 | | **5.22** | **1.71** | **15.99** |
| 70-97 | | 1.32 | 1.09 | 1.61 | **1.34** | **1.08** | **1.67** | 2.53 | 0.51 | 12.61 | **5.57** | **1.08** | **28.81** | 1.78 | | 1.19 | 2.66 | **1.87** | **1.21** | **2.91** | 1.90 | | 0.42 | | 8.53 | | 4.40 | 0.90 | 21.58 |
| **BMI** | |  |  |  |  |  |  |  |  |  |  |  |  |  | |  |  |  |  |  |  | |  | |  | |  |  |  |
| <18.5, underweight | | 0.71 | 0.54 | 0.93 | 0.76 | 0.58 | 1.00 | 3.51 | 0.32 | 38.89 | 2.79 | 0.25 | 31.52 | 0.66 | | 0.34 | 1.29 | 0.76 | 0.39 | 1.49 | 3.78 | | 1.49 | | 9.59 | | **6.10** | **2.15** | **17.26** |
| 18.5-24.9, normal | | 1 |  |  | 1 |  |  | 1 |  |  | 1 |  |  | 1 | |  |  | 1 |  |  | 1 | |  | |  | | 1 |  |  |
| >25, overweight | | 1.42 | 1.24 | 1.62 | **1.33** | **1.16** | **1.53** | 4.28 | 0.83 | 22.09 | 5.15 | 0.99 | 26.93 | 1.58 | | 1.19 | 2.11 | **1.42** | **1.05** | **1.91** | 1.18 | | 0.50 | | 2.78 | | 0.95 | 0.39 | 2.30 |
| **Substance use** | |  |  |  |  |  |  |  |  |  |  |  |  |  | |  |  |  |  |  |  | |  | |  | |  |  |  |
| None | | 1 |  |  | 1 |  |  | 1 |  |  | 1 |  |  | 1 | |  |  | 1 |  |  | 1 | |  | |  | | 1 |  |  |
| Cigarettes | | 2.01 | 1.63 | 2.47 | **1.69** | **1.36** | **2.11** | 3.89 | 0.75 | 20.20 | 3.58 | 0.59 | 21.61 | 3.07 | | 2.06 | 4.60 | **2.41** | **1.57** | **3.70** | 1.62 | | 0.19 | | 13.54 | | 0.88 | 0.10 | 7.68 |
| Alcohol | | 1.29 | 0.91 | 1.85 | 1.06 | 0.74 | 1.52 | - | - | - | - | - | - | 2.41 | | 1.25 | 4.66 | 1.85 | 0.94 | 3.65 | 8.32 | | 1.65 | | 41.89 | | **6.15** | **1.13** | **33.62** |
| Betel quid | | 1.66 | 0.64 | 4.32 | 1.39 | 0.53 | 3.64 | - | - | - | - | - | - | 1.90 | | 0.24 | 15.11 | 1.53 | 0.19 | 12.31 | - | | - | | - | | - | - | - |
| Cigarettes+Alcohol | | 2.52 | 1.88 | 3.38 | **2.08** | **1.53** | **2.83** | - | - | - | - | - | - | 3.70 | | 2.15 | 6.38 | **2.83** | **1.60** | **5.01** | 23.46 | | 7.41 | | 74.29 | | **13.71** | **3.93** | **47.90** |
| Cigarettes+Betel quid | | 2.32 | 1.46 | 3.69 | **1.73** | **1.08** | **2.79** | - | - | - | - | - | - | 6.21 | | 3.12 | 12.35 | **4.28** | **2.09** | **8.78** | 9.83 | | 1.15 | | 83.98 | | 4.72 | 0.51 | 43.56 |
| Alcohol+Betel quid | | 2.49 | 1.05 | 5.93 | 1.92 | 0.80 | 4.60 | - | - | - | - | - | - | - | | - | - | - | - | - | 36.06 | | 3.93 | | 330.64 | | **25.35** | **2.50** | **256.79** |
| Cigarettes+Alcohol+Betel quid | | 2.27 | 1.62 | 3.17 | **1.74** | **1.23** | **2.46** | 5.90 | 0.68 | 51.21 | 6.86 | 0.64 | 73.11 | 5.69 | | 3.39 | 9.56 | **3.98** | **2.29** | **6.93** | 59.00 | | 21.48 | | 162.03 | | **29.23** | **9.35** | **91.39** |
| **Diabetes** | |  |  |  |  |  |  |  |  |  |  |  |  |  | |  |  |  |  |  |  | |  | |  | |  |  |  |
| No | | 1 |  |  | 1 |  |  | 1 |  |  | 1 |  |  | 1 | |  |  | 1 |  |  | 1 | |  | |  | | 1 |  |  |
| Yes | | 1.17 | 0.97 | 1.42 | 0.97 | 0.79 | 1.19 | 1.08 | 0.13 | 8.82 | 1.59 | 0.18 | 13.80 | 1.09 | | 0.72 | 1.67 | 0.74 | 0.48 | 1.16 | 0.87 | | 0.26 | | 2.90 | | 0.77 | 0.22 | 2.75 |
| **Hypertension** | |  |  |  |  |  |  |  |  |  |  |  |  |  | |  |  |  |  |  |  | |  | |  | |  |  |  |
| No | | 1 |  |  | 1 |  |  | 1 |  |  | 1 |  |  | 1 | |  |  | 1 |  |  | 1 | |  | |  | | 1 |  |  |
| Yes | | 1.22 | 1.06 | 1.41 | 1.08 | 0.93 | 1.27 | - | - | - | - | - | - | 1.57 | | 1.17 | 2.11 | 1.37 | 0.99 | 1.90 | 0.77 | | 0.31 | | 1.91 | | 0.65 | 0.25 | 1.70 |
| **Cancer family history** | |  |  |  |  |  |  |  |  |  |  |  |  |  | |  |  |  |  |  |  | |  | |  | |  |  |  |
| No | | 1 |  |  | 1 |  |  | 1 |  |  | 1 |  |  | 1 | |  |  | 1 |  |  | 1 | |  | |  | | 1 |  |  |
| Yes | | 1.01 | 0.87 | 1.16 | 1.06 | 0.92 | 1.23 | 2.86 | 0.71 | 11.48 | 3.69 | 0.89 | 15.25 | 0.99 | | 0.72 | 1.36 | 1.09 | 0.79 | 1.52 | 1.09 | | 0.48 | | 2.47 | | 1.19 | 0.50 | 2.82 |
| Abbreviations: cOR, crude odds ratio; aOR, adjusted odds ratio; CI, confidence interval | | | | | | | | | | | | | |  |  |  |  |  |  |  |  |  |  |  |  |  |  |  |  |
| ^1^Using esophagus normal as reference category. Adjusted odds ratio were adjusted for all variables listed in this table. | | | | | | | | | | | | | | | | | | | | |  | |  | |  |  |  |  |  |
| Bold indicates statistical significance. | | | | | | | | | | | | | |  |  |  |  |  |  |  |  |  |  |  |  |  |  |  |  |
